# Effect of Vitamin K-Mediated PXR Activation on Drug-Metabolizing Gene Expression in Human Intestinal Carcinoma LS180 Cell Line

**DOI:** 10.3390/nu13051709

**Published:** 2021-05-18

**Authors:** Halima Sultana, Ayaka Kato, Ai Ohashi, Rie Takashima, Tomoko Katsurai, Shoko Sato, Masafumi Monma, Yusuke Ohsaki, Tomoko Goto, Michio Komai, Hitoshi Shirakawa

**Affiliations:** 1 Laboratory of Nutrition, Graduate School of Agricultural Science, Tohoku University, 468-1 Aramaki Aza Aoba, Aoba-ku, Sendai 980-8572, Japan; sultana.halima.d4@tohoku.ac.jp (H.S.); a.kato@g-mail.tohoku-univeristy.jp (A.K.); a.ohashi@g-mail.tohoku-univerity.jp (A.O.); r.takashima@g-mail.tohoku-univeristy.jp (R.T.); t.katsurai@g-mail.tohoku-univeristy.jp (T.K.); satosho@iam.u-tokyo.ac.jp (S.S.); m.monma@g-mail.tohoku-univeristy.jp (M.M.); yusuke.ohsaki.a4@tohoku.ac.jp (Y.O.); tgoto@mgu.ac.jp (T.G.); mkomai@m.tohoku.ac.jp (M.K.); 2 International Education and Research Center for Food Agricultural Immunology, Graduate School of Agricultural Science, Tohoku University, 468-1 Aramaki Aza Aoba, Aoba-ku, Sendai 980-8572, Japan

**Keywords:** pregnane X receptor, vitamin K, isoprenoids, drug–nutrient interaction

## Abstract

The pregnane X receptor (PXR) is the key regulator of our defense mechanism against foreign substances such as drugs, dietary nutrients, or environmental pollutants. Because of increased health consciousness, the use of dietary supplements has gradually increased, and most of them can activate PXR. Therefore, an analysis of the interaction between drugs and nutrients is important because altered levels of drug-metabolizing enzymes or transporters can remarkably affect the efficiency of a co-administered drug. In the present study, we analyzed the effect of vitamin K-mediated PXR activation on drug metabolism-related gene expression in intestine-derived LS180 cells via gene expression studies and western blotting analyses. We demonstrated that menaquinone 4 (MK-4), along with other vitamin Ks, including vitamin K_1_, has the potential to induce *MDR1* and *CYP3A4* gene expression. We showed that PXR knockdown reversed MK-4-mediated stimulation of these genes, indicating the involvement of PXR in this effect. In addition, we showed that the expression of *MDR1* and *CYP3A4* genes increased synergistically after 24 h of rifampicin and MK-4 co-treatment. Our study thus elucidates the importance of drug–nutrient interaction mediated via PXR.

## 1. Introduction

Vitamin K (VK) is a fat-soluble vitamin that plays a significant role in blood coagulation and bone formation. The parent structure of VK has 2-methyl-1,4-naphthoquinone, also known as menadione or VK_3_, as its basic skeleton. VK is classified into two groups, namely, VK_1_ with phytyl side chains, also known as phylloquinone, and VK_2_, a group of compounds with isoprenoid side chains of various lengths, commonly known as menaquinones (MKs) [1,2]. VK_1_ is predominantly contained in green leafy vegetables and vegetable oils, whereas VK_2_ is mainly produced by microorganisms, and therefore it is found in fermented foods such as natto (fermented soybeans) and cheese. VK acts as a cofactor of γ-glutamyl carboxylase (GGCX), which modifies the reaction during carboxylation of the γ-position of specific glutamic acid residues of VK-dependent (VKD) proteins to produce γ-carboxy glutamic acid residue (Gla). The enzyme activity of GGCX is important for the activation of blood coagulation factors II, VII, IX, and X, protein S, protein C, and protein Z. VKD proteins such as osteocalcin, matrix Gla protein, and growth arrest-specific protein 6 play important roles in modulating bone metabolism, arterial calcification, and cell proliferation. Because of its beneficial effect, MK-4, a member of the VK_2_ family, has been used as a therapeutic agent for osteoporosis in many Asian countries [3,4]. Apart from maintaining blood and bone homeostasis, VK has been reported to regulate more extensive processes such as inflammation, testosterone production, cancer progression, neuroprotection, bile metabolism, and type 2 diabetes [5,6,7,8,9,10].

The pregnane X receptor (PXR encoded by *NR1I2* gene, also known as steroid and xenobiotic receptor or SXR), the master regulator of xenobiotic metabolism, was identified in 1998 as a member of the nuclear receptor (NR) superfamily of ligand-activated transcription factors. The biotransformation process and detoxification of drug/xenobiotics mostly occur in the liver and intestine, where PXR is predominantly expressed [11,12,13]. PXR has common structural features of an NR: An N-terminal domain, a DNA-binding domain, a hinge region, and a ligand-binding domain (LBD) [14]. However, because of the presence of enlarged, flexible, and hydrophobic LBD, PXR can be activated by various substances. PXR LBD contains an insert of about 60 residues, which is not found in the LBD of other NRs [15]. Because of this special structural feature, LBD of PXR can change its shape, thereby allowing various ligands, not only drugs but also nutrients and their metabolites, to bind based on their nature [16].

PXR is activated by xenobiotics, dietary substances, and exogenous and endogenous substances like bile acids (BAs) [16,17,18,19,20]. The reactions of drug/xenobiotic metabolism can be divided into three phases, namely, phase I (hydroxylation), phase II (conjugation), and phase III (transport). Several genes involved in these phases are directly regulated by PXR [21]. Among the enzymes encoded by these genes, the most important are those belonging to cytochrome P450 3A family (CYP3A), because they are involved in metabolizing approximately 50% of prescribed drugs [22,23]. Within the CYP3A subfamily, CYP3A4 plays a significant role in the oxidative metabolism of drugs because of its abundance in the liver and intestine as well as its broad substrate specificity [11,24]. The multi-drug resistance 1 (*MDR1*) gene is present in the human intestine, and is a direct target of PXR [25]. The *MDR1* gene encodes P-glycoprotein 1, which is a multi-functional broadly-selective drug efflux pump. However, recently, numerous studies have revealed that PXR regulates not only drug metabolism but also many physiological functions such as inflammation, bone homeostasis, lipid and BA homeostasis, vitamin D metabolism, energy homeostasis, and diseases like cholestasis, inflammatory bowel disorders, and cancer [19].

In 2003, Tabb MM. et al. demonstrated that MK-4 directly acts as a ligand of PXR, thereby triggering the formation of heterodimers with 9-*cis*-retinoid acid receptor. Subsequently, the complex binds to PXR response elements (PXRE) within the regulatory regions of target genes [4,26]. Thus, transcriptionally activated PXR mediates the binding of coactivators with PXR, thus modulating the expression of genes involved in bone homeostasis [4]. Ichikawa, T. et al. further evaluated the role of PXR activation in the regulation of gene expression during bone homeostasis using MK-4 and Rif, a well-known ligand of human PXR [27]. The study showed that MK-4-mediated activation of PXR regulates both osteoblastogenesis and osteoclastogenesis [27]. Later, the PXR-mediated effect of MK-4 was demonstrated using human hepatocellular carcinoma cells, which revealed that MK-4 suppresses cell proliferation and motility by acting both as a cofactor of GGCX and as a ligand to enhance PXR activation, thereby preventing the occurrence and recurrence of hepatocellular carcinoma [28]. Recently, another group of researchers demonstrated that both MK-4 and lithocholic acid (LCA), a secondary BA produced by intestinal microbes, could activate PXR, resulting in the subsequent expression of *CYP3A4* and *CYP2C9* genes in human pluripotent stem cells-derived and isolated fetal hepatocytes. Additionally, the study demonstrated that both LCA and MK-4 could drive the metabolic maturation of human embryonic stem cell-derived hepatocytes [29]. However, to our knowledge, the interaction between VK and PXR in intestinal cells has not yet been analyzed.

Furthermore, PXR activation with a ligand can increase the expression of *CYP3A* or *MDR1* genes, which are involved in drug metabolism, potentially resulting in adverse health issues for the patient. For example, St. John’s wort is an antidepressant and a well-known ligand of PXR that has been reported to interact with different types of medicines ‒ the immunosuppressant cyclosporin, the HIV proteinase inhibitor indinavir, the anticoagulant warfarin, and oral contraceptives [23,30]. Therefore, it is necessary to investigate the effects of VK on PXR-mediated activation of *CYP3A4* and *MDR1* genes, because VK is consumed as a nutritional supplement.

We hypothesized that VK-mediated PXR activation may affect the metabolism of other drugs. In this study, we investigated the changes in gene expression mediated by MK-4-activated PXR in human intestinal carcinoma cells (LS180), and compared the effects of changes in the side chain structure. Finally, we analyzed the effects of interactions between nutrients and drugs and revealed that nutrient consumption alongside drugs may affect drug metabolism, thereby having an adverse effect on the patient.

## 2. Materials and Methods

### 2.1. Materials

The human intestinal epithelial cell line, LS180, was purchased from American Type Culture Collection (Manassas, VA, USA) and cultured in Eagle’s minimum essential medium (MEM, Sigma-Aldrich, St. Louis, MO, USA) supplemented with 10% fetal calf serum, antibiotics (100 units/mL penicillin and 100 mg/mL streptomycin), sodium pyruvate (1 mM), and non-essential amino acid solution (Invitrogen, Carlsbad, CA, USA) in a humidified atmosphere at 37 °C with 95% air and 5% CO_2_. MK-4 was kindly obtained from Nisshin Pharma Inc., Tokyo, Japan. MK-7 and VK_1_ were purchased from Wako Pure Chemical Industries (Osaka, Japan). Geraniol, farnesol, geranylgeraniol and Rif were purchased from Sigma-Aldrich. The antibodies against MDR1 and α-tubulin were obtained from Cell Signaling Technology Inc. (Danvers, MA, USA) and Sigma-Aldrich, respectively.

### 2.2. Cell Proliferation Assay

LS180 cells were seeded onto 96-well plates and cultured for 24 h. The cells were treated with VK analogs with different side chain structures or Rif and ethanol (EtOH) for 48 h. Cell proliferating activity was estimated using the Premix WST-1 Cell Proliferation Assay System (Takara Bio, Otsu, Japan) to determine the toxicity of each compound used in this experiment. The absorbance was measured at 450 nm and 630 nm using the Microplate reader Model 680 XR (Bio-Rad Laboratories, Hercules, CA, USA).

### 2.3. RNA Isolation and Quantitative RT-PCR Analysis

LS180 cells were cultured for 16 h. Thereafter, the medium was changed to a fresh medium supplemented with MK-4 and Rif or control vehicle (EtOH) at 0.1% *v*/*v* following incubation for an appropriate length of time. The cells were washed twice with phosphate-buffered saline (PBS). Total RNA was isolated using ISOGEN reagent (Nippon Gene, Tokyo, Japan). Overall, 4 µg of total RNA was denatured with 2.5 µM oligo-dT primer (GE Healthcare, Tokyo, Japan) and 0.5 mM dNTP (GE Healthcare) at 65 °C for 5 min. The RNA was incubated in 20 µL reaction buffer [50 mM Tris-HCl at pH 8.3, 75 mM potassium chloride, 3 mM magnesium chloride, and 5 mM dithiothreitol (DTT)] containing 50 U of reverse transcriptase (SuperscriptⅢ, Invitrogen) and 20 U of RNase inhibitor (RNaseOUT, Invitrogen) at 50 °C for 60 min. An aliquot of synthesized cDNA was used as a template for following quantitative PCR. We used primers listed in Table 1 for amplification of the target cDNAs in the TB Green Premix Ex Taq solution (Takara Bio). Relative expression levels of mRNA were normalized to those of eukaryotic translation elongation factor 1 α1 (*EEF1A1*) mRNA.

### 2.4. Western Blotting

LS180 cells were collected by scraping in lysis buffer [50 mM Tris-HCl at pH 7.5, 150 mM NaCl, 0.1% sodium dodecyl sulfate (SDS), and 5 mM ethylenediamine tetraacetic acid] containing phosphatase and protease inhibitors (Roche Applied Science, Mannheim, Germany). Proteins were denatured in gel-loading buffer, and approximately 15 µg proteins were resolved on 10–20% SDS polyacrylamide gel (Wako Pure Chemical Industries, Osaka, Japan) via electrophoresis. Separated proteins from the gel were transferred onto polyvinyldifluoride membrane (Millipore, Billerica, MA, USA). The membrane was blocked for 2 h in TBS-T buffer (10 mM Tris-HCl at pH 7.5, 150 mM NaCl, and 0.1% Tween 20) containing 5% skimmed milk. The membrane was then incubated overnight with blocking buffer containing the antibody against MDR1, followed by incubation for 1 h with horseradish peroxidase (HRP)-tagged secondary antibody. The membrane was incubated with the antibody against α-tubulin for 1 h, followed by HRP-tagged secondary antibody. The immunoreactive band was detected using the Immobilon Western Detection Reagent (Millipore) and visualized using the LAS-4000 mini luminescent image analyzer (Fujifilm, Tokyo, Japan). Relative protein levels were measured by normalizing the levels with that of α-tubulin.

### 2.5. Transient Transfection and Luciferase Assay

Luciferase reporter gene was constructed following procedure. The 5′-flanking region of human *MDR1* gene (−7864 to −7817), which contains PXRE, was amplified using KOD-plus DNA polymerase (Toyobo, Osaka, Japan) in PCR and ligated into a pGL4.12 luciferase plasmid (Promega, Madison, WI, USA) that has the promoter sequence of herpes simplex virus thymidine kinase gene. LS180 cells were seeded onto 12-well plates and cultured for 16 h. The cells were then transiently transfected with the luciferase reporter plasmid (MDR1/pGL4-TK) and pCH110-β-galactosidase transfection control plasmid using the FuGENE HD Transfection Reagent (Roche Applied Science) in additive-free MEM for 24 h. The cultured medium was replaced with fresh MEM containing MK-4 or Rif and EtOH, and the cells were incubated for another 24 h. The cells were washed with PBS and lysed in Passive Lysis Buffer (Promega). Following centrifugation at 12,000× *g* at 4 °C for 2 min, luciferase activity in the supernatant was determined using the Luciferase Assay Reagent (Promega). A β-Galactosidase assay was performed using the Galacto-star System (Applied Biosystems). Chemiluminescence was detected using Luminescenser-MCA AB-2250 (Atto Co., Tokyo, Japan). Reporter gene activity was normalized to β-galactosidase activity.

### 2.6. RNA Interference

LS180 cells were seeded onto 60 mm dishes for 16 h before transfection. Stealth small-interfering RNAs (siRNAs, 30 pmol; Invitrogen) for human PXR were transfected into the cells using Lipofectamine RNAi MAX (Invitrogen) with Opti-MEM-Ⅰ medium (Invitrogen) for 24 h according to the manufacturer’s protocol. After the transfection, the medium was replaced with MEM supplemented with MK-4 or Rif, and the cells were incubated for another 24 h. Then total RNA was isolated from cells.

### 2.7. High-Performance Liquid Chromatography (HPLC) Analysis of VK in LS180 Cells

LS180 cells were seeded onto 12-well plates and cultured for 16 h. The medium was replaced with additive-free MEM supplemented with VK analogs, and the cells were incubated for an appropriate length of time. The cells were washed twice with PBS and suspended in 1 mL PBS. VK was extracted from the suspension using 5 mL of 66% 2-propanol and 6 mL of *n*-hexane containing 13.6 ng of MK-5, and the amount of VK was analyzed using a fluorescence-HPLC system, as previously described [31]. VK_1_, MK-4, and MK-7 concentrations were calculated relative to the fluorescence intensity of MK-5, which was used as an internal standard.

### 2.8. Statistical Analysis

Data are presented as mean ± standard error of the mean. Statistical analysis was carried out using Student’s *t*-test or one-way analysis of variance (ANOVA), followed by Dunnett’s test or the Tukey–Kramer test, or a two-way ANOVA followed by the Tukey–Kramer test. All statistical analyses were considered significant at *p* < 0.05.

## 3. Results

### 3.1. Effect of MK-4 on Drug Metabolism-Related Gene Expression in LS180 Cells

**Figure 1:**
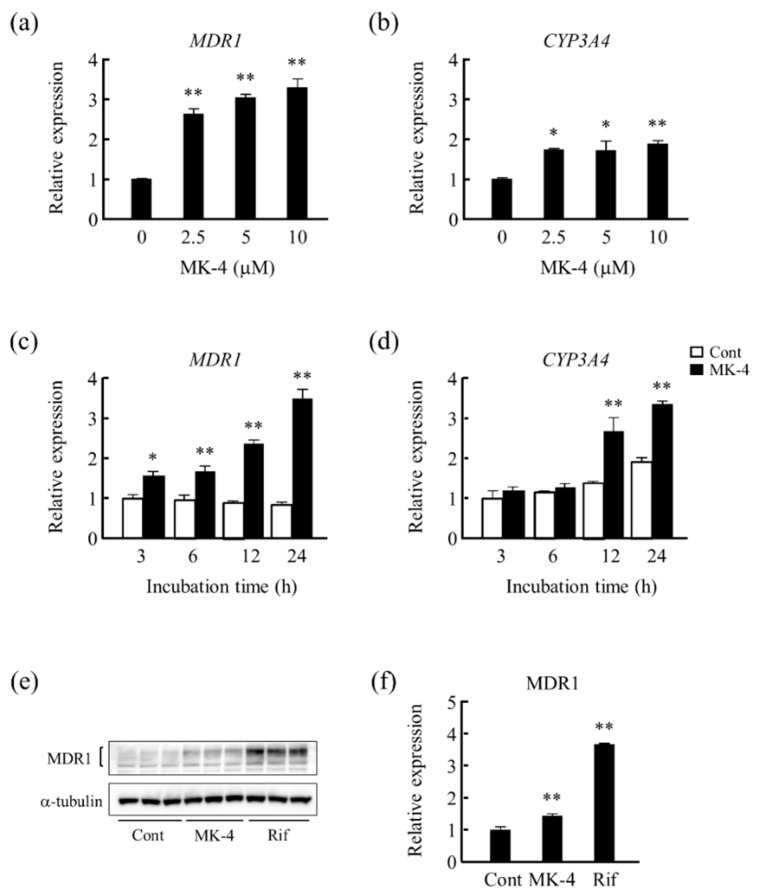
Effect of menaquinone-4 (MK-4) on drug metabolism-related gene expression in LS180 cells. The effects of different (2.5–10 μM) concentrations of MK-4 for 24 h on (**a**) *CYP3A4* and (**b**) *MDR1* mRNA levels in LS180 cells were analyzed. The effects of MK-4 on (**c**) *MDR1* and (**d**) *CYP3A4* mRNA levels were measured after 3, 6, 12, and 24 h of treatment. mRNA levels were measured using quantitative RT-PCR. Relative mRNA levels of the genes were normalized to that of eukaryotic translation elongation factor 1 α1 mRNA. (**e**,**f**) Effects of MK-4 and rifampicin (Rif) treatment on MDR1 protein levels were analyzed using western blotting after 24 h of treatment. Data are expressed as mean ± standard error of the mean, *n* = 3, * *p* < 0.05, ** *p* < 0.01 vs. MK-4 (0 μM) (**a**,**b**), or control (Cont) (**c**,**d**,**f**). *MDR1*, multi-drug resistance; *CYP3A4*, cytochrome P450 3A subfamily 4.

First, we tested the effect of MK-4 on drug metabolism-related gene expression in LS180 cells. We found that *MDR1* and *CYP3A4* mRNA levels were significantly increased at each of the concentrations tested (2.5–10 μM) (Figure 1a,b). Upon treatment with 10 μM of MK-4, *MDR1* mRNA level was significantly increased at each of the time points (Figure 1c), whereas *CYP3A4* mRNA level was significantly increased after 12 and 24 h of the treatment (Figure 1d). Furthermore, upon treating LS180 cells with MK-4 or Rif for 24 h, MDR1 protein levels were significantly increased when compared with those of the control (Figure 1e,f). The extent of the enhancement of mRNA levels by MK-4 did not correspond to that of protein levels in LS180 cells because protein levels of MDR1 could be regulated by post transcriptional mechanisms in this cell.

### 3.2. Analysis of PXR Transcriptional Activation by MK-4

**Figure 2:**
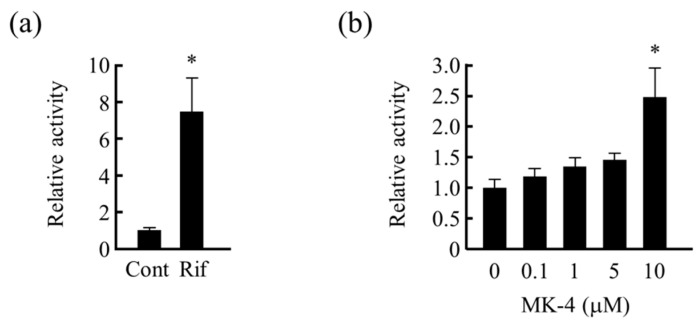
Effect of rifampicin (Rif) and menaquinone-4 (MK-4) on MDR1/pGL4-TK reporter gene activity. To determine the transcriptional activation of PXR by MK-4, the effects of (**a**) Rif and (**b**) different concentrations of MK-4 on MDR1/pGL4-TK reporter gene activity in LS180 cells were analyzed. Data are expressed as the mean ± standard error of the mean, *n* = 3, * *p* < 0.05 vs. control (Cont) (**a**) or MK-4 (0 μM) (**b**). *MDR1*, multi-drug resistance.

As MK-4 induces the expression of drug metabolism-related genes, and MK-4 is reported to be a ligand of human PXR in osteoblasts and hepatocellular carcinoma, we analyzed whether PXR is involved in this gene expression-inducing effect of MK-4. We examined the effects of MK-4 on reporter activity using reporter gene, including the transcriptional regulatory region of human *MDR1* gene (MDR1/pGL4-TK), in LS180 cells. The reporter gene activity was significantly increased after Rif and MK-4 (10 μM) treatment (Figure 2a,b).

**Figure 3:**
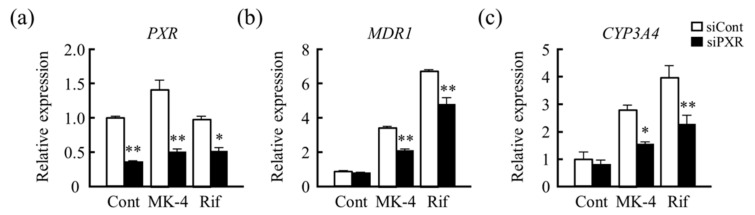
Effect of PXR knockdown on drug metabolism-related genes. To investigate the regulation of PXR in drug metabolism-related gene expression induced by menaquinone-4 (MK-4), the effect of siRNA-mediated knockdown of PXR on the mRNA levels of (**a**) *PXR*, (**b**) *MDR1*, and (**c**) *CYP3A4* after 24 h of MK-4 treatment was analyzed. The effect was compared with that of rifampicin (Rif) treatment. Relative mRNA levels were normalized to those of eukaryotic translation elongation factor 1 α1 mRNA. Data are expressed as the mean ± standard error of the mean, n = 3, * *p* < 0.05, ** *p* < 0.01 vs. control (siCont). *PXR*, pregnane X receptor; *MDR1*, multi-drug resistance; *CYP3A4*, cytochrome P450 3A subfamily 4.

Furthermore, PXR knockdown using siRNA resulted in PXR mRNA levels less than 50% those of the control, MK-4, and Rif groups (Figure 3a). The stimulatory effects of MK-4 and Rif on *MDR1* and *CYP3A4* mRNA levels were found to be reduced significantly (Figure 3b,c) because of knockdown of PXR. These results suggest that PXR is involved in inducing the expression of *MDR1* and *CYP3A4* genes by MK-4 in LS180 cells.

### 3.3. Effects of Different Forms of VK on Drug Metabolism-Related Gene Expression in LS180 Cells

**Figure 4:**
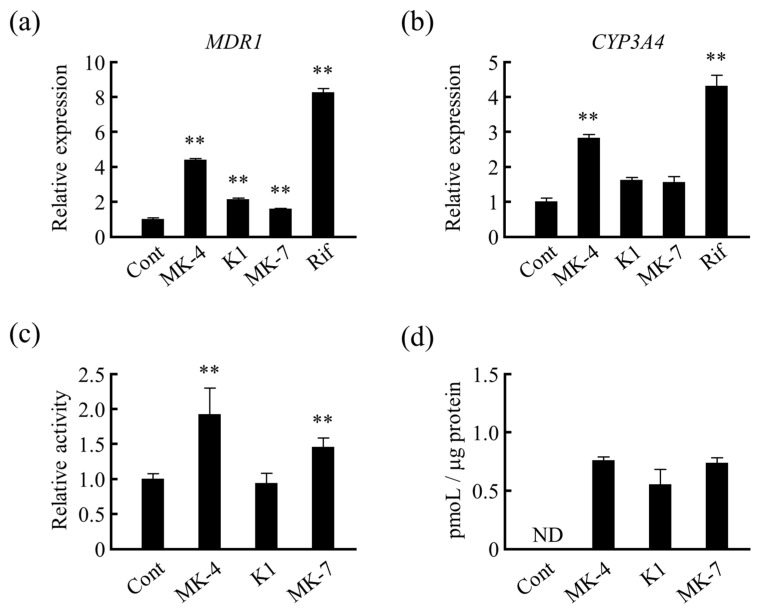
Effects of different forms of vitamin K (VK) on drug metabolism-related gene expression. Effects of treatment with different forms of VK for 24 h on (**a**) *CYP3A4* and (**b**) *MDR1* mRNA levels in LS180 cells were analyzed. Relative mRNA levels were normalized to that of eukaryotic translation elongation factor 1 α1 mRNA. (**c**) Effect of different forms of VK on MDR1/pGL4-TK reporter gene activity was analyzed. (**d**) Uptake of VK content by LS180 cells after 24 h treatment with different forms of VK was measured using HPLC. Data are expressed as the mean ± standard error of the mean, *n* = 3, ** *p* < 0.01 vs. control (Cont). MK-4, menaquinone-4; K1, vitamin K_1_; MK-7, menaquinone-7; Rif, rifampicin; HPLC; high-performance liquid chromatography. *MDR1*, multi-drug resistance; *CYP3A4*, cytochrome P450 3A subfamily 4.

Next, we compared the mRNA levels of drug-metabolizing genes after treating LS180 cells with different forms of VK. We found that *MDR1* mRNA levels were significantly increased by not only MK-4 but also other VK homologs (Figure 4a). Similarly, *CYP3A4* mRNA expression was significantly increased by MK-4 treatment when compared with that of the control, and an increasing trend was observed when treated with VK_1_ and MK-7 (Figure 4b). Additionally, MDR1/pGL4-TK reporter gene activity was significantly increased by MK-4 and MK-7 but not by VK_1_ (Figure 4c). Here, it can be noted that the order of ability of VKs for upregulating the *MDR1* gene expression in reporter gene assay did not follow the order of ability of VKs to induce the expression of actual mRNA (Figure 4a) because reporter gene assay reflects the result of only synthesis (transcription) whereas mRNA expression reflects the combined results of synthesis and degradation of mRNA. However, the results of HPLC analyses showed that there was no difference in the uptake amount of different forms of VK by the cells after 24 h of the treatment (Figure 4d). Thus, the results suggest that the activation of PXR target genes was reduced possibly because of the reduced ability of ligands to activate PXR and not because of the cellular concentrations of VK_1_ and MK-7.

**Figure 5:**
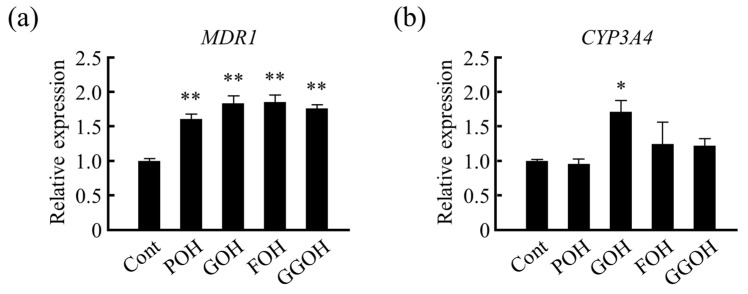
Effects of isoprenoid side chain structures on drug metabolism-related gene expression. (**a**) *MDR1* and (**b**) *CYP3A4* mRNA levels were measured using quantitative RT-PCR after 24 h of treatment with different types of isoprenoid structures. Relative mRNA levels were normalized to that of eukaryotic translation elongation factor 1 α1 mRNA. Data are expressed as the mean ± standard error of the mean, *n* = 3, * *p* < 0.05, ** *p* < 0.01 vs. control (Cont). *MDR1*, multi-drug resistance; *CYP3A4*, cytochrome P450 3A subfamily 4; POH, phytol; GOH, geraniol; FOH, farnesol; GGOH, geranyl geraniol.

Further, we analyzed the effects of isoprenoid side chain structures of VK on drug metabolism-related gene expression. We analyzed the effects of phytol (POH; a side chain of VK_1_), geraniol (GOH) with 2 isoprene units, farnesol (FOH) with 3 isoprene units, and geranyl geraniol (GGOH) with 4 isoprene units on drug metabolism-related gene expression in LS180 cells. *MDR1* mRNA levels were increased in all of the treatment groups when compared with that of the control group (Figure 5a). However, the effect of partially saturated structure, POH, was lower than that of other isoprenoid structures. Similar effects were observed on *CYP3A4* mRNA levels (Figure 5b). Therefore, these results suggest that the side chain structure of VK is important for PXR ligand activity.

### 3.4. Effects of Drug–Nutrient Interaction on Drug Metabolism-Related Gene Expression

**Figure 6:**
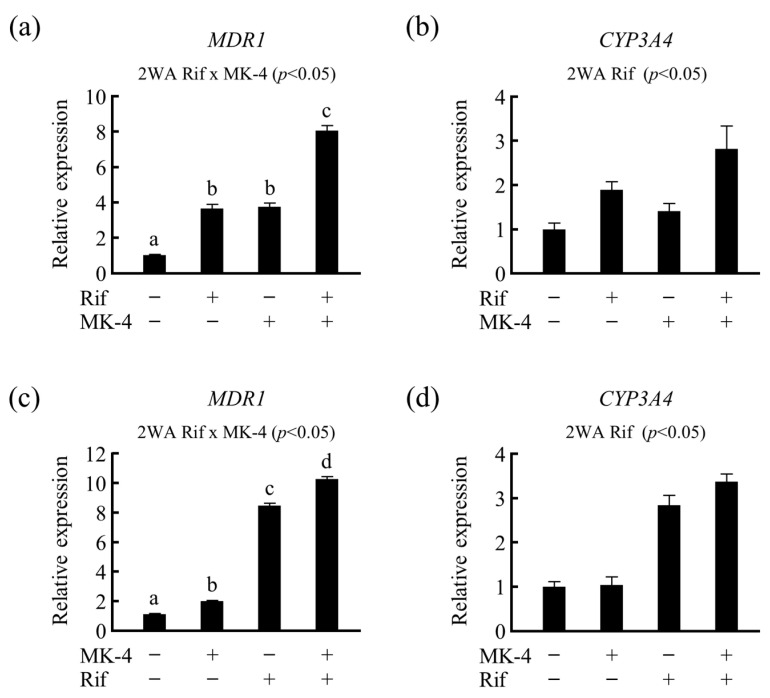
Effects of drug–nutrient interaction on drug metabolism-related gene expression. Effects of Rif pretreatment on the mRNA levels of (**a**) *MDR1* and (**b**) *CYP3A4* in LS180 cells were analyzed after 24 h of menaquinone-4 (MK-4) treatment. Similarly, the effects of MK-4 pretreatment on the mRNA levels of (**c**) *MDR1* and (**d**) *CYP3A4* in LS180 cells were analyzed after 24 h of Rif treatment. mRNA levels were measured using quantitative RT-PCR. Relative mRNA levels were normalized to that of eukaryotic translation elongation factor 1 α1 mRNA. Data are expressed as the mean ± standard error of the mean, *n* = 3. Data were analyzed using two-way analysis of variance. Different letters indicate significant difference. *MDR1*, multi-drug resistance; *CYP3A4*, cytochrome P450 3A subfamily 4; Rif, rifampicin; RT-PCR, reverse transcription polymerase chain reaction.

To investigate whether the expression of drug metabolism-related genes is altered by nutrients in the presence of drugs, LS180 cells were treated with MK-4 after pretreatment with 50 µM of Rif, and the expression of drug-metabolizing genes was analyzed. We found that *MDR1* mRNA level was notably increased owing to the synergistic action of Rif and MK-4 when compared with that of the control as well as single treatment of MK-4 or Rif (Figure 6a). A similar effect was observed for *CYP3A4* mRNA levels; however, it was not statistically significant (Figure 6b). Additionally, after pretreating the cells with MK-4 for 24 h, Rif treatment synergistically elevated *MDR1* mRNA levels (Figure 6c), whereas the treatment only slightly increased *CYP3A4* mRNA levels (Figure 6d). Thus, these results suggest that VK may modulate the effect of prescribed medications that act as a ligand for PXR.

## 4. Discussion

In the present study, we demonstrated that the expression of drug metabolism-related genes was significantly affected by MK-4 treatment through PXR activity in LS180 cells. Additionally, other VK analogs and their side chain structural moieties enhanced *MDR1* mRNA levels, suggesting that isoprenoid side chain structure is important for PXR ligand activity. We found that the co-treatment of LS180 cells with nutrient (MK-4) and drug (Rif) increased the expression level of drug-metabolizing genes when compared with that of the drug treatment alone.

Oral administration of drugs is the most common route employed for treatment purposes. To become bioavailable, an orally ingested medicine passes progressively from the gastrointestinal track through the gut wall and finally through the liver to the systemic circulation. The small intestine is considered to be the most important extrahepatic site of drug biotransformation because it is the first site where xenobiotics enter the metabolic system; thus, a relatively high concentration of the ingested drug reaches the intestine. Consequently, there is a high possibility of a clinically significant amount of drug interacting with food metabolites in the intestine. Moreover, the large surface area of the small intestine allows the absorption of drug/xenobiotics for subsequent metabolism [32,33]. A study on paired human tissues reported that MDR1 and CYP3A4 protein levels in the intestine were higher than that in the liver, indicating the importance of drug metabolism in the intestine [34].

LS180 is a human intestinal colon carcinoma cell line that is suitable for investigating the regulation of *CYP3A4* and *MDR1* expression by PXR, because this cell line not only presents some of the characteristics of the small intestine but also expresses PXR endogenously [32,35,36]. Moreover, Rif, a prototypical ligand of PXR, could induce *CYP3A* and *MDR1* expression by activating PXR in human intestinal cells [32,37,38]. Consistent with this result, several studies have reported that pregnenolone 16α-carbonitrile, a typical ligand of rodent PXR, enhanced *Mdr1a/1b* mRNA, as well as the protein levels in all segments of the rodent intestine [39,40,41,42,43,44].

In the current study, we found that the expression of *CYP3A4* and *MDR1* induced by MK-4 was concentration- and time-dependent. We examined the effect of MK-4 on reporter activity using reporter construct containing the transcriptional regulatory region of human *MDR1* gene. A significant increase in reporter activity was observed after treatment with MK-4. To analyze the involvement of PXR in these stimulatory effects, we examined the effect of PXR knockdown, using siRNA, on drug metabolism-related gene expression. When siRNA was transfected into LS180 cells, a significant decrease in the stimulatory effect of MK-4 was observed via reduced *MDR1* and *CYP3A4* mRNA levels. These results suggest that PXR is involved in the transcriptional regulation of *MDR1* and *CYP3A4* genes by MK-4.

Furthermore, we tested the effects of VK with different side chains on the expression of drug-metabolizing genes. We found that *MDR1* mRNA levels were significantly increased by all forms of VK tested, whereas *CYP3A4* mRNA levels were significantly increased only by MK-4. The difference in the gene expression-inducing effect by different types of VK analogs has been considered to be related to the side chain structure of vitamins and their ability as ligands to activate PXR. Probably, PXR is activated more by relatively short side chains, such as of MK-4, than structures with long side chains such as of MK-7 in LS180 cells. Suhara Y. et al. hypothesized that the modification of the side chain structures of natural VK would play a more important role in biological activities and synthesized a number of new VK analogues [45,46,47]. The studies have investigated PXR mediated gene transcription to evaluate the effects of the side chains of VK. However, the authors reported that VK_1_ could not activate PXR in hepatocellular carcinoma (HepG2) cells. They demonstrated that the ability of VK analog to activate PXR transcriptionally was decreased with the decreasing number of double bonds in the side chain [45]. The contradictory result could be due to the different cell lines used in these studies. However, both studies suggest that the side chain structure is important for the ligand activity of PXR. Furthermore, we found that the effect of MK-4, VK_1_, and MK-7 on drug metabolism-related gene expression (Figure 4) are different from that of the corresponding side chain moieties (Figure 5), possibly because the binding affinity to PXR depends on the length of the side chain part as well as the fitting of the naphthoquinone part of VK analogues to the LBD of PXR [46].

Moreover, in our study, as compared to *CYP3A4* expression, *MDR1* expression level was found to be more affected by VK treatment. This can be explained by ligand and promoter selective regulation of PXR-mediated gene expression that has been reported by Masuyama H. et al. [48]. Different ligands of PXR can regulate PXR binding to different response elements, thereby modulating co-activator interactions in the promoter regions of target genes [48]. For example, paclitaxel and cisplatin are anticancer drugs which can induce PXR binding more strongly to PXRE in *MDR1* promoter region than that in *CYP3A4* promoter region [48]. In addition to this, tissue-specific induction of PXR target genes has been reported. This could be because different levels of co-activators and co-repressors can be expressed following a tissue-specific manner, thereby differentially affecting the activation of NRs [48]. For example, tocotrienol (T3), a vitamin E homologue, was reported to be a ligand for PXR in HepG2 and LS180 cells and showed cell-specific induction of PXR target genes [49]. It was found that T3 upregulates the expression of *CYP3A4* but not *MDR1* in primary hepatocytes, whereas the opposite effect was observed in intestinal LS180 cells [49]. The study demonstrated that NR co-repressor (NCoR) plays an important role in cell-specific gene expression regulation by PXR. The unbound PXR interacts with NCoR, whereas T3 disrupts this interaction only partially in LS180 cells because of the relatively high expression of NCoR in LS180 cells when compared with that in HepG2 cells [49].

Finally, we analyzed the drug–nutrient interaction in LS180 cells. Recently, the global dietary supplement market has expanded with the increasing focus on health [50]. It has been reported that 45.8% of Japanese adults aged 55–75 years old take dietary supplements on a weekly or daily basis [51]. Among college students, it is 16.8%, whereas 37.5% of children are provided with vitamin and mineral supplements in Japan [50,52]. According to these studies, 7.5% of college students and 3.6% of children were reported to experience some adverse effects because of these supplements. Bailey et al. described that about half of the USA population (44% of males and 53% of females) reportedly take dietary supplements, and a majority of them take one supplement on a daily basis [53]. According to this study, multivitamins/multiminerals are the most frequently used dietary supplements (33%) in the USA. Therefore, it is important to clarify the underlying drug–nutrient interactions to avoid the risk of potential adverse effect as much as possible. Hence, we investigated the effects of MK-4 and Rif co-treatment in LS180 cells and analyzed their combined effect on drug metabolism-related gene expression. We found that MK-4 potentiated the effects of Rif on PXR target gene expression synergistically.

## 5. Conclusions

In conclusion, this is the first study investigating the stimulatory effects of MK-4 on *CYP3A4* and *MDR1* expression via PXR activation in human intestine-derived LS180 cells. We demonstrated for the first time that not only MK-4 but also VK_1_ and other MKs have the potential to induce *CYP3A4* and *MDR1* expression in LS180 cells. Furthermore, we demonstrated that Rif (drug) and VK (nutrient) co-treatment affects drug metabolism, thereby suggesting that MK-4 consumption may cause alterations in the metabolism of PXR-activating prescribed medications, which may have serious consequences if patients consume both drugs and nutrients at the same time.

## Figures and Tables

**Table 1:** Nucleotide sequences of primers for amplification of the target cDNA in quantitative RT-PCR.

Gene	Forward (5′–3′)	Reverse (5′–3′)
*PXR*	TGGACGCTCAGATGAAAACCTT	CACTTGGCAGCTTCTTCCCTC
*MDR1*	CCCATCATTGCAATAGCAGG	TGTTCAAACTTCTGCTCCTGA
*CYP3A4*	TGGTGATGATTCCAAGCTATGCTC	AATGCAGTTTCTGGGTCCACTTC
*EEF1A1*	GATGGCCCCAAATTATTGAAG	GGACCATGTCAATGGCAG

*PXR*, pregnane X receptor; *MDR1*, multi-drug resistance; *CYP3A4*, cytochrome P450 3A subfamily 4; *EEF1A1*, eukaryotic translation elongation factor 1 α1.

## Data Availability

Data is contained within the article.
